# Association between personality type and patient-reported outcomes (PRO) in patients with atrial fibrillation

**DOI:** 10.1186/s12872-024-04098-1

**Published:** 2024-08-22

**Authors:** Qing Yan, Jiaqi Liang, Yide Yuan, Yuan Li, Jiali Fan, Wenhuan Wu, Pan Xu, Qunrang Wang, Jiahong Xue

**Affiliations:** 1 Department of Cardiovascular Medicine, The Second Affiliated Hospital of Xi’an Jiaotong University, 157 West Five Road, Xi’an, Shaanxi 710004 China; 2 Department of Cardiovascular Medicine, Zichang People’s Hospital, Zichang, Shaanxi 717300 China; 3 Department of Ultrasound, Xijing Hospital, Shaanxi, 710032 China; 4 Department of Cardiovascular Medicine, the First Affiliated Hospital of Shaanxi University of Traditional Chinese Medicine, Xianyang, Shaanxi 712000 China; 5 Xinjiang Medical University Affiliated Cancer Hospital, 789 East Suzhou Street, Urumqi, Xinjiang, 830000 China

**Keywords:** Atrial fibrillation, Personality types, Patient-reported outcomes (PRO), Quality of life (QoL), Anxiety, Depressed

## Abstract

**Background:**

Atrial Fibrillation (AF) is known to be associated with a negative emotional state. Patient-reported outcomes (PROs) are important tools for evaluating the endpoints of AF management. This study aims to examine the correlation between personality types and PROs in patients with AF.

**Methods:**

All included subjects were newly diagnosed with AF fewer than one month, and their personality types were assessed using the Eysenck Personality Questionnaire (EPQ). Quality of life (QoL) was measured using the Atrial Fibrillation Effect on Quality of Life (AFEQT) questionnaire. Anxiety and depression were assessed using the General Anxiety Scale (GAD-7) and the 9-item Patient Health Questionnaire (PHQ-9), respectively. We constructed stepwise linear regression analyses for factors related to the QoL and emotional state in patients with AF.

**Results:**

A total of 531 AF patients completed the survey and were categorized into four groups based on their personality types. Of these patients (mean age: 67.12 ± 10.93 years, 50.28% male), 357 (67.23%) had paroxysmal AF, and 16.95% (*n* = 90) had a sanguine personality. Compared to patients with other personality types, those with a sanguine personality had the highest average AFEQT scores (*P* < 0.001) and the lowest scores of GAD-7 and PHQ-9 scales (*P* < 0.05). Furthermore, multiple linear regression analyses suggested that sanguine personality was also independently associated with better QoL and emotional states (*P* < 0.05).

**Conclusion:**

There is a significant association between the personality types and PROs in AF patients.

## Background

Atrial Fibrillation (AF) is a severe disorder of atrial electrical activity. It results in the disruption of regular and orderly atrial electrical activity, replaced by rapid and chaotic fibrillation waves. AF increases the risk of stroke, heart failure and death, significantly impairing the quality of life (QoL) in patients [1, 2]. In 2017, a study reported 37.6 million cases of AF, resulting in 287,200 deaths [3, 4]. The incidence of AF in China is also significant. Over the past 11 years, there has been a 20-fold increase in AF incidence and a 13-fold rise in AF-related strokes [5]. This trend is further compounded by the rapid aging of the population.

It’s widely known that the effectiveness of AF treatment can be assessed through validated, disease-specific patient-reported outcomes (PROs). It has been recommended by various national and international organizations and healthcare associations [6]. These PROs encompass the severity of AF symptoms, AF-related quality of life, functional status, and emotional well-being (anxiety and depression) [7]. The Atrial Fibrillation Effect on Quality of Life (AFEQT) questionnaire is recognized as one of the most effective methods for evaluating PROs in the context of AF [8, 9]. Previous studies have indicated that depression and anxiety are independent predictors of AF recurrence [10–12], and are linked to reduced quality of life in individuals with AF [13–15].

However, few studies have focused on the impact of various personality types, such as choleric personality, melancholic personality, phlegmatic personality, and sanguine personality, on the occurrence and recurrence of AF. Some studies indicate that individuals with a D-type personality are prone to experience negative emotions, which are associated with sympathetic activation [16] and may adversely affect the QoL in AF patients [17]. Additionally, personality types are believed to be closely intertwined with depression and anxiety, potentially exerting an influence on individuals’ quality of life [18]. Hence, we initiated this exploratory study to investigate the association between personality types and PROs in individuals with atrial fibrillation. It would be of great significance to the clinician that psychological counseling is provided to patients with particular personality types in the early stage of AF to alleviate anxiety and depression and improve their QoL.

## Materials and methods

### Study design and population

This study is a multi-center cross-sectional study that adhered to ethical standards outlined in the World Medical Association Declaration of Helsinki. Approval for the study was obtained from the Ethics Review Board of the Second Affiliated Hospital of Xi’an Jiaotong University (NO. 2018.188) and written informed consent was obtained from all participants. A total of 754 eligible patients with atrial fibrillation from three different centers were screened between October 2021 and April 2023. Of those, 407 were from the Second Affiliated Hospital of Xi’an Jiaotong University, 230 were from the First Affiliated Hospital of Shaanxi University of Traditional Chinese Medicine, and 117 were from Zichang People’s Hospital.

The inclusion criteria for participants were: (1) newly diagnosed paroxysmal or persistent AF fewer than one month prior to enrollment, and (2) aged between 18 and 80 years. Exclusion criteria included: (1) severe structural heart diseases (such as valvular heart disease, dilated cardiomyopathy, and hypertrophic cardiomyopathy), (2) severe liver or kidney dysfunction, neoplastic diseases, or other life-threatening conditions, (3) presence of mental illness or long-term use of anti-anxiety and depression drugs, (4) inability to understand and complete the questionnaire, and (5) incomplete questionnaire information (personality types cannot be assessed).

### Assessment of QoL in AF patients

The Atrial Fibrillation Effect on Quality of Life (AFEQT) questionnaire was employed to evaluate the influence of AF on the QoL of all participants. The AFEQT questionnaire consists of 20 items organized into four distinct domains: symptoms, daily activities, treatment concerns, and treatment satisfaction (Cronbach’s α = 0.82 in this study). Each domain is assessed using a 1–7 Likert scale, and a composite score is derived from the initial three domains. Both the overall scores and subscale scores are scaled from 0 to 100, where a score of 0 signifies complete impairment, while a score of 100 indicates no impairment [19].

### Assessment of personality types in AF patients

**Fig. 1:**
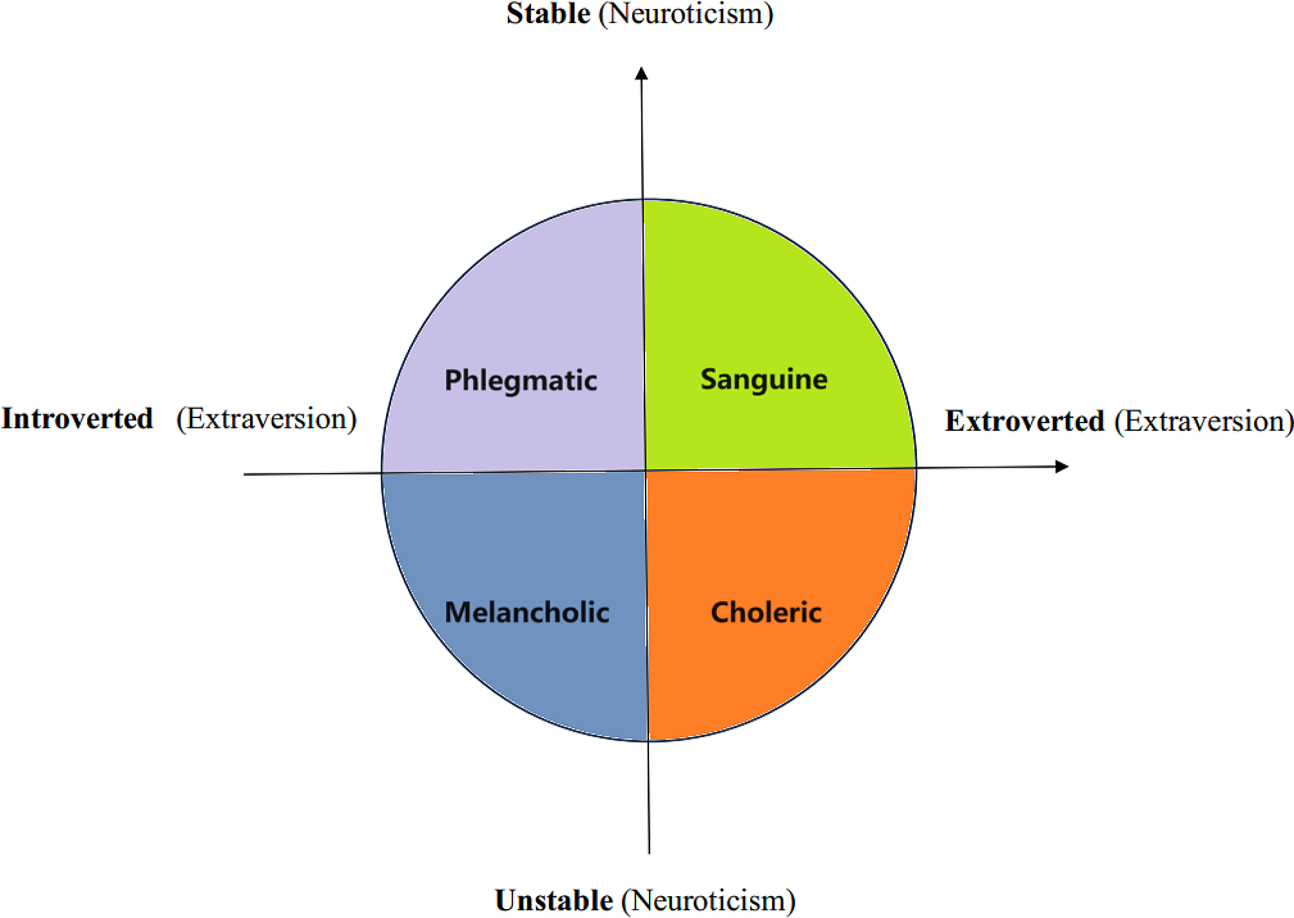
Characterization of four personality types

The Eysenck Personality Questionnaire (EPQ), developed by the British psychologist H. J. Eysenck, is a self-report scale used to assess personality types. In the revised Chinese version, the number of items was reduced from 107 to 88, encompassing four subscales: internal and external propensity (E), neuroticism (N), psychoticism (P), and lying (L) [20]. Eysenck then constructed a personality structure map using extraversion and neuroticism as two primary dimensions, with extraversion as the horizontal axis and neuroticism as the vertical axis, ranging from emotionally stable to emotionally unstable. This model revealed that the personality types delineated by the E and N dimensions correspond to the four classical personality types: choleric, melancholic, phlegmatic, and sanguine (Fig. 1) [21]. In this study, subjects were categorized into these four personality types based on their scores on the E and N dimensions, with Cronbach’s alpha of 0.74 and 0.81 in E and N dimensions indicating good reliability.

### Assessment of emotional state in AF patients

The anxiety and depression statuses of the patients were evaluated using the GAD-7 (Cronbach’s α = 0.92 in this study) and PHQ-9 scales (Cronbach’s α = 0.81 in this study).

The GAD-7 scale, a self-rated anxiety questionnaire comprising seven items, exhibits strong reliability (Cronbach’s α = 0.89) and validity among both primary care patients and the general population. Each item is evaluated on a four-point scale, resulting in a total score ranging from 0 (No anxiety) to 21 (The worst anxiety) [22].

The PHQ-9 scale, consisting of nine items, is a depression questionnaire designed to detect potential cases of depression and evaluate the intensity of symptoms experienced in the preceding two weeks. This depression assessment tool has demonstrated strong reliability across various medical conditions (Cronbach’s α = 0.86–0.89). Scores on this scale can range from 0 (No depression) to 27 (The worst depression) [23].

### Statistical analysis

Continuous variables were expressed as mean ± standard deviation, and categorical variables were expressed as percentages. The differences between groups were analyzed using either 1-way analysis of variance or the Kruskal-Wallis rank sum test. The Chi-square test was employed to analyze categorical variables. Univariate and stepwise linear regression were used to analyze the factors associated with the QoL and emotional state in AF patients. A significance level of *P* < 0.05 was used to determine statistical significance. All statistical analyses were conducted using SPSS version 18.

## Results

### Patient cohort and baseline characteristics

**Fig. 2:**
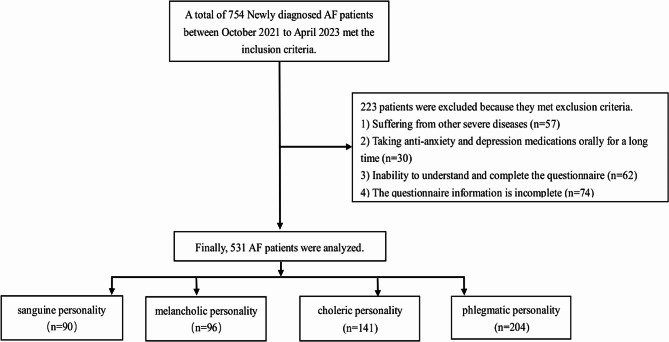
Subject screening procedure

A total of 754 patients with AF were initially included in this study. However, 223 individuals were excluded due to not meeting the criteria for inclusion. Specifically, 57 subjects had other severe diseases, 30 patients orally took anti-anxiety and depression medications, 62 participants couldn’t comprehend and complete the questionnaire, and 74 individuals had incomplete questionnaire information. Ultimately, 531 patients (age: 67.12 ± 10.93, 50.28% male) successfully completed the study (Fig. 2). Among these patients, 357 (67.23%) had paroxysmal AF, while 174 (32.77%) had persistent AF.

These 531 participants were categorized into four groups based on the EPQ. Specifically, there were 90 (16.95%) subjects with a sanguine personality, 96 (18.08%) with a melancholic personality, 141 (26.55%) with a choleric personality, and 204 (38.42%) with a phlegmatic personality. No significant differences were observed among participants with different personality types in terms of the incidence of CHD, diabetes, hyperlipidemia, gender, BMI, educational level, CHA2DS2-VASc scores, and the use of antiarrhythmic drugs and anticoagulants (*P* > 0.05). However, significant differences could be seen in the experience of hypertension, age, and AF type across the different personality groups (*P* < 0.05) (Table 1). Notably, AF patients with a phlegmatic personality were generally older.

**Table 1:** Baseline characteristics of AF patients

Variables	All Subjects (*N* = 531)	Choleric Personality (*N* = 141)	Melancholic Personality (*N* = 96)	Phlegmatic Personality (*N* = 204)	Sanguine Personality (*N* = 90)	F / X²	*P*
Age: (years)	67.12 ± 10.93	63.66 ± 11.07	66.84 ± 10.68	71.93 ± 9.32	61.93 ± 10.34	9.386	<0.001
Female, N (%)	264 (49.72)	63 (44.68)	42 (43.75)	126 (61.76)	33 (36.67)	6.925	0.074
Height: (cm)	166.43 ± 7.74	169.04 ± 7.90	164.91 ± 7.15	164.28 ± 7.96	168.83 ± 5.78	5.264	0.002
Weight: (kg)	65.16 ± 10.86	67.50 ± 10.01	65.35 ± 12.03	62.60 ± 10.86	67.10 ± 10.05	2.370	0.072
BMI: (kg/m²)	23.42 ± 3.21	23.61 ± 3.24	23.91 ± 3.20	23.02 ± 3.33	23.49 ± 2.90	0.648	0.585
Smoking, N (%)	102 (19.21)	48 (34.04)	12 (12.50)	24 (11.76)	18 (20.00)	10.032	0.018
Alcoholism, N (%)	57 (10.73)	30 (21.28)	3 (3.13)	9 (4.41)	15 (16.67)	11.324	0.010
CHD, N (%)	285 (53.67)	66 (46.81)	45 (46.88)	132 (64.71)	42 (46.67)	5.407	0.144
Hypertension, N (%)	312 (58.76)	96 (68.09)	57 (59.38)	93 (45.59)	66 (73.33)	9.189	0.027
Diabetes, N (%)	156 (29.38)	36 (25.53)	21 (21.88)	51 (25.00)	48 (53.33)	3.82	0.282
Hyperlipemia, N (%)	132 (24.86)	42 (29.79)	30 (31.25)	30 (14.71)	30 (33.33)	6.217	0.102
AADs, N (%)						7.554	0.580
Amiodarone	177 (33.33)	57 (40.43)	21 (21.87)	63 (30.87)	36 (40.00)		
Propafenone	30 (5.65)	9 (6.38)	6 (6.25)	12 (5.88)	3 (3.33)		
β-blockers	261 (49.15)	60 (42.55)	57 (59.38)	111 (54.41)	33 (36.67)		
Amiodarone + β-blockers	63 (11.87)	15 (10.64)	12 (12.50)	18 (8.84)	18 (20.00)		
Anticoagulant, N (%)						2.648	0.449
Rivaroxaban	492 (92.66)	135 (95.74)	87 (90.63)	192 (94.12)	78 (86.67)		
Dabigatran	39 (7.34)	6 (4.26)	9 (9.37)	12 (5.88)	12 (13.33)		
Degree of education, N (%)						9.821	0.056
Below primary school	111 (20.90)	24 (17.02)	12 (12.50)	69 (33.82)	6 (6.67)		
Junior high-senior high	291 (54.80)	78 (55.32)	57 (59.38)	99 (48.53)	57 (63.33)		
Bachelor degree or above	129 (24.30)	39 (27.66)	27 (28.12)	36 (17.65)	27 (30.00)		
Paroxysmal AF, N (%)	357 (67.23)	120 (85.11)	72 (75.00)	90 (44.12)	75 (83.33)	27.714	<0.001
CHA2DS2-VASc Scores >2), N (%)	249 (46.89)	69 (48.94)	39 (40.62)	111 (54.41)	30 (33.33)	4.342	0.227
AFEQT Scores							
Total	66.40 ± 18.01	62.17 ± 17.79	58.51 ± 16.43	64.75 ± 15.91	85.19 ± 11.10	18.059	<0.001
Symptoms	68.05 ± 20.32	63.72 ± 22.09	60.37 ± 18.35	67.60 ± 18.96	84.03 ± 13.45	9.676	<0.001
Daily Activities	62.36 ± 20.15	57.87 ± 19.03	53.84 ± 19.04	59.66 ± 17.51	84.58 ± 12.39	20.263	<0.001
Treatment Concerns	72.71 ± 19.42	69.68 ± 21.28	64.06 ± 17.91	72.01 ± 18.02	88.24 ± 11.42	10.339	<0.001

BMI: Body mass index; CHD: Coronary heart disease; AF: Atrial fibrillation; AADs: Antiarrhythmic drugs

### AFEQT scores for subjects with different personality types

**Fig. 3:**
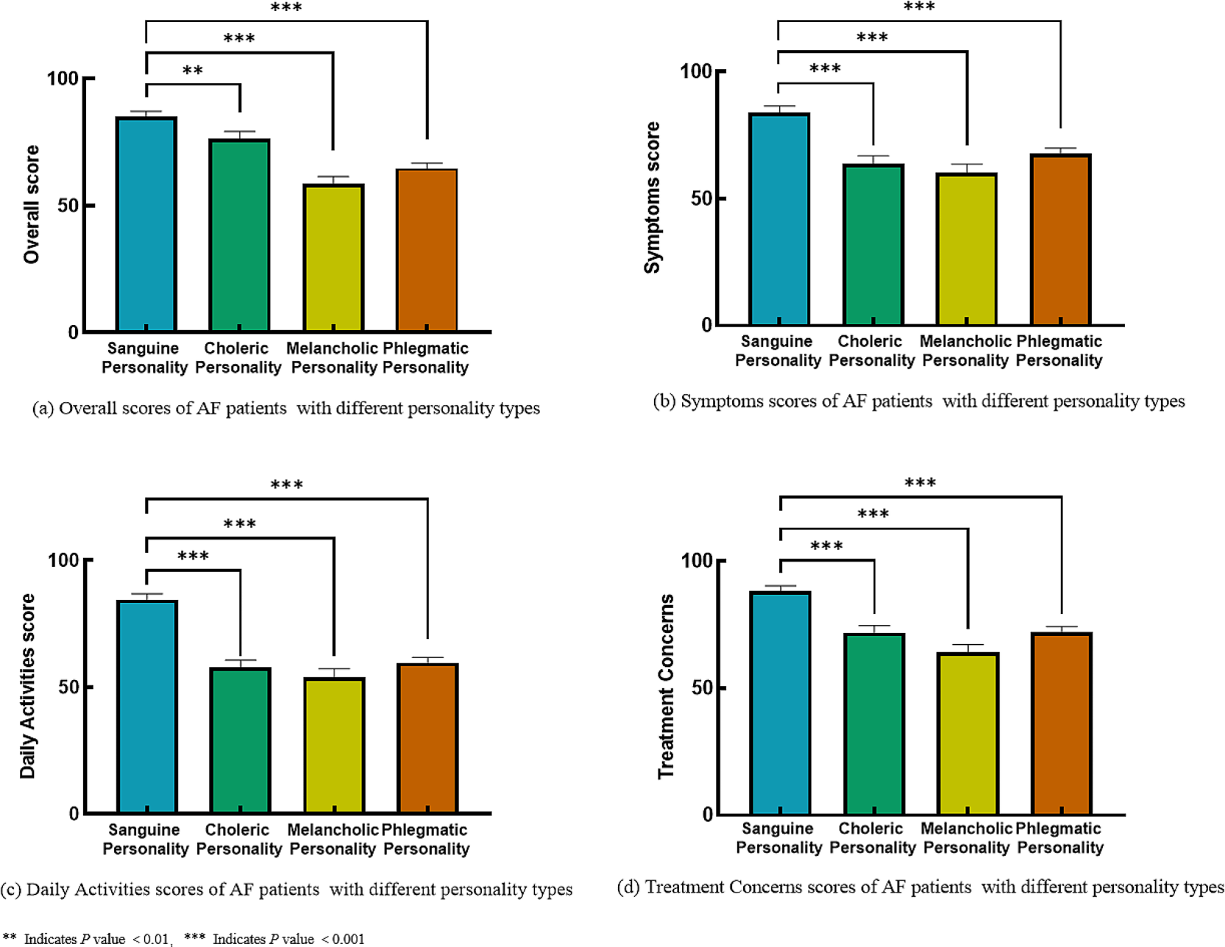
AFEQT overall scores and sub-scales score in AF patients with different personality types. (**a**): The total scores of AFEQT questionnaire in AF patients with different personality types. (**b**) (**c**) (**d**): The scores of the AFEQT sub-scale in AF patients with different personality types

Table 1 displays the AFEQT scores for subjects with various personality types. Patients with a sanguine personality had the highest overall mean AFEQT score (85.19 ± 11.09). The scores for choleric, melancholic, and phlegmatic patients were 62.17 ± 17.79, 58.51 ± 16.43, and 64.75 ± 15.91, respectively (*P* < 0.001). The scores of the AFEQT subscale were further examined. Patients with a sanguine personality scored the highest in all subscales of the AFEQT (Symptoms: 84.03 ± 13.45, Daily Activities: 84.58 ± 12.39, Treatment Concerns: 88.24 ± 11.42), and there were significant differences compared to other groups (All *P* values<0.05) (Fig. 3).

### Factors influencing AFEQT scores in AF patients

As shown in Table 2, the results of the univariate analyses suggested that age, CHD history and personality types displayed different AFEQT scores (*P*<0.05 for all). A multivariable linear regression analysis was employed to pinpoint the determinants of the overall AFEQT score in patients diagnosed with AF. (refer to Table 2). Notably, the analysis demonstrated that individuals exhibiting choleric, melancholic, and phlegmatic personality types had lower mean AFEQT scores by 8.893, 26.818, and 25.329 points, respectively, when contrasted against those with a sanguine personality. These findings underscore the differential impact of personality types on the quality of life among AF patients.

**Table 2:** Stepwise multiple linear regression analysis of AFEQT scores in AF patients

Variables	Univariate analysis		Multivariable analysis	
	β (95%CI)	*P*	β (95%CI)	*P*
**Age** (>65 years)	-0.268 (-0.511~-0.026)	0.030		
**Gender** (Female)	-3.815 (-9.142 ~ 1.512)	0.159		
**BMI** (>24 kg/m²)	-0.060 (-0.898 ~ 0.778)	0.888		
**Smoking**	-0.774 (-7.572 ~ 6.025)	0.823		
**Alcoholism**	-1.393 (-10.044 ~ 7.258)	0.751		
**CHD**	-6.045 (-11.340~-0.749)	0.026		
**Hypertension**	3.710 (-1.730 ~ 9.123)	0.178		
**Diabetes**	5.204 (-0.625 ~ 11.034)	0.080		
**Hyperlipemia**	-4.433 (-10.595 ~ 1.730)	0.158		
**Catheter ablation**	0.802 (0.408, 1.562)	0.547		
**AF type** (Persistent AF)	3.966 (-1.710 ~ 9.643)	0.170		
**Degree of education**		0.833		
Below primary school	Reference	——		
Junior high-senior high	1.969 (-4.573 ~ 8.510)			
Bachelor degree or above	0.976 (-7.030 ~ 8.982)			
Personality Traits*				
Sanguine Personality	Reference	——	Reference	——
Choleric Personality	-23.018 (-30.328~-15.708)	<0.001	-8.893 (-16.077, -1.709)	0.016
Melancholic Personality	-26.679 (-34.629~-18.730)	<0.001	-26.818 (-34.628, -19.008)	< 0.001
Phlegmatic Personality	-20.435 (-27.291~-13.579)	<0.001	-25.329 (-33.258, -17.400)	< 0.001

BMI: Body mass index; CHD: Coronary heart disease; AF: Atrial fibrillation; AADs: Antiarrhythmic drugs

### Emotional state in subjects with different personality types

**Fig. 4:**
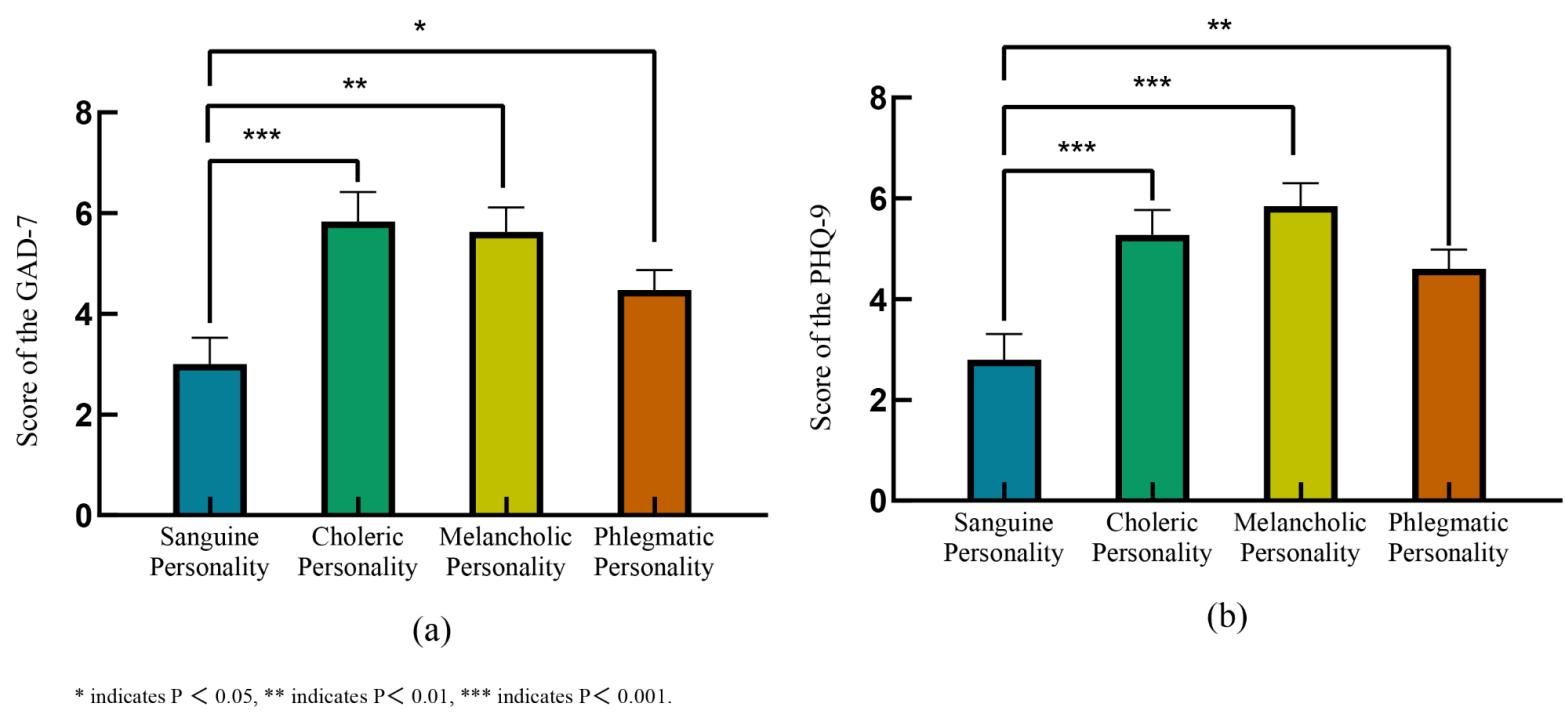
Emotional states of AF patients with different personality types. (**a**): Scores of the GAD-7 scale in patients with different personality types. (**b**): Scores of the PHQ-9 scale in patients with different personality types

As illustrated in Fig. 4(a), a significant disparity in the scores of the GAD-7 scale was observed amongst the distinct personality groups (*P* = 0.002). Specifically, patients with a sanguine personality displayed the lowest mean scores (3 ± 2.89) on the GAD-7 scale. Conversely, the mean scores for patients with choleric, melancholic, and phlegmatic personalities were 5.83 ± 4.06, 5.63 ± 2.79, and 4.47 ± 3.28, respectively. Similarly, there were pronounced variations in the the scores of PHQ-9 scale among different personality types (F = 28.400, *P* = 0.001) as shown in Fig. 4(b). Once again, the sanguine personality group had the lowest mean scores on the PHQ-9 scale (2.80 ± 2.76), while the mean scores for choleric, melancholic, and phlegmatic patients were 5.28 ± 3.35, 5.84 ± 2.59, and 4.60 ± 3.12, respectively.

### Factors influencing emotional state in AF patients

As delineated in Table 3, the outcomes of the multivariable linear regression analysis unveiled a compelling association between personality types and GAD-7 scores among patients diagnosed with AF, with statistical significance (*P* < 0.001). After adjusting for potential confounding variables, the analysis highlighted that, relative to the sanguine personality group, patients identified within the choleric (β = 2.830, 95% CI: 1.278–4.382, *P* < 0.001), melancholic (β = 2.625, 95% CI: 0.937–4.313, *P* = 0.002), and phlegmatic (β = 1.471, 95% CI: 0.015–2.926, *P* = 0.048) personality types manifested notably higher scores on the GAD-7 scale. This finding suggests a greater degree of anxiety severity in these respective personality groups compared to those with a sanguine personality.

**Table 3:** Stepwise multiple linear regression analysis of anxiety in AF patients

Variables	Univariate analysis		Multivariable analysis	
	β (95%CI)	*P*	β (95%CI)	*P*
**Age** (>65 years)	1.092 (0.604, 1.977)	0.770		
**Gender** (Female)	1.611 (0.889, 2.919)	0.116		
**BMI** (>24 kg/m²)	0.680 (0.372, 1.242)	0.210		
**Smoking**	0.730 (0.342, 1.557)	0.416		
**Alcoholism**	0.996 (0.384, 2.583)	0.993		
**CHD**	0.689 (0.380, 1.247)	0.218		
**Hypertension**	0.881 (0.484, 1.604)	0.678		
**Diabetes**	0.929 (0.486, 1.776)	0.823		
**Hyperlipemia**	0.795 (0.400, 1.580)	0.513		
**Catheter ablation**	0.815 (0.418, 1.587)	0.547		
**AF type** (Persistent AF)	0.694 (0.368, 1.309)	0.259		
**Degree of education**		0.483		
Below primary school	Reference			
Junior high-senior high	1.561 (0.753, 3.238)	0.231		
Bachelor degree or above	1.449 (0.596, 3.523)	0.413		
Personality Traits*				
Sanguine Personality	Reference	——	Reference	——
Choleric Personality	6.750 (2.201, 20.705)	0.001	2.830 (1.278, 4.382)	< 0.001
Melancholic Personality	21.667 (5.860, 80.112)	< 0.001	2.625 (0.937, 4.313)	0.002
Phlegmatic Personality	3.095 (1.054, 9.092)	0.040	1.471 (0.015, 2.926)	0.048

BMI: Body mass index; CHD: Coronary heart disease; AF: Atrial fibrillation; AADs: Antiarrhythmic drugs

Furthermore, an investigation into the determinants of depression levels in patients with AF was conducted, with the results meticulously outlined in Table 4. The multivariable linear regression analysis revealed a significant association between depression severity and personality types (*P* < 0.05). Upon controlling for potential confounding variables, the analysis demonstrated that, in comparison to the sanguine personality cohort, individuals within the choleric (β = 2.478, 95% CI: 1.096–3.860, *P* = 0.001), melancholic (β = 3.046, 95% CI: 1.543–4.549, *P* < 0.001), and phlegmatic (β = 2.004, 95% CI: 0.697–3.310, *P* = 0.003) personality types exhibited markedly higher scores on the PHQ-9 scale. This indicates a greater burden of depression in these personality groups relative to those with a sanguine personality.

**Table 4:** Stepwise multiple linear regression analysis of depression in AF patients

Variables	Univariate analysis		Multivariable analysis	
	β (95%CI)	*P*	β (95%CI)	*P*
**Age** (>65 years)	0.749 (0.411, 1.363)	0.344		
**Gender** (Female)	1.871 (1.023, 3.423)	0.042		
**BMI** (>24 kg/m²)	0.725 (0.394, 1.334)	0.301		
**Smoking**	0.693 (0.318, 1.506)	0.354		
**Alcoholism**	0.772 (0.289, 2.065)	0.606		
**CHD**	1.072 (0.589, 1.950)	0.820		
**Hypertension**	1.093 (0.596, 2.005)	0.773		
**Diabetes**	0.795 (0.411, 1.540)	0.497		
**Hyperlipemia**	2.184 (1.093, 4.364)	0.027		
**Catheter ablation**	1.080 (0.553, 2.109)	0.821		
**AF type** (Persistent AF)	1.587 (0.842, 2.990)	0.153	1.113 (0.207, 2.018)	0.016
**Degree of education**		0.255		
Below primary school	Reference			
Junior high-senior high	1.323 (0.614, 2.851)	0.475		
Bachelor degree or above	0.708 (0284, 1.768)	0.460		
**Personality Traits**				
Sanguine Personality	Reference	——	Reference	——
Choleric Personality	14.500 (3.835, 54.820)	< 0.001	2.478 (1.096, 3.860)	0.001
Melancholic Personality	15.000 (3.732, 60.285)	< 0.001	3.046 (1.543, 4.549)	< 0.001
Phlegmatic Personality	4.600 (1.261, 16.782)	0.021	2.004 (0.697, 3.310)	0.003

BMI: Body mass index; CHD: Coronary heart disease; AF: Atrial fibrillation; AADs: Antiarrhythmic drugs

Additionally, the analysis uncovered that patients experiencing persistent AF reported higher levels of depression than those with paroxysmal AF (β = 1.113, 95% CI: 0.207–2.018, *P* = 0.016), further highlighting the multifaceted impact of AF type on mental health.

## Discussion

The study revealed that there is a strong link between the personality type of patients with AF and their PROs. Patients with a sanguine personality exhibited the best QoL and the lowest scores of the GAD-7 and PHQ-9 scales. Even after adjusting for clinical background through multivariable regression analysis, personality types remained independently associated with QoL and emotional state of AF patients. Our results emphasize the importance of considering personality as a fundamental aspect of AF patient evaluation and management in clinical practice.

It is widely recognized that personality types and psychiatric factors have a correlation with cardiovascular disease risk. In the 1980s, a survey indicated that type A behavior was associated with an elevated risk of heart disease, independent of serum cholesterol, high blood pressure, and smoking [24]. Additionally, a case-control study by Anna et al. in 2005 suggested a higher incidence of AF among patients with type A behavior patterns [25]. Moreover, patients complicated with type D personality are at a higher risk of adverse cardiovascular outcomes [26, 27]. Additional studies have also demonstrated an association between negative emotions, such as depression, and an increased risk of coronary heart disease (CHD) [28], as well as AF [29]. Conversely, a recent large-scale individual patient data meta-analysis found no significant effect of type D personality on cardiac and all-cause mortality in patients with CAD and heart failure (HF). Nevertheless, type D personality still poses an increased risk for adverse events in patients with CAD [30]. In our study, personality types were classified as choleric, melancholic, phlegmatic, and sanguine personalities using the EPQ. We know that Type D personality is often characterized by negative affectivity (dysphoria, anxiety, irritability) and social inhibition (inhibited behavior during social interaction), which is similar to the characteristics of melancholic personality (introversion and instability) [31]. Although we did not specifically examine the influence of different personality types on the occurrence and progression of AF, we did find that personality had significant effects on the PROs of AF patients. Our results showed that patients with different personality types exhibited differences in self-conscious symptoms, symptom severity, and emotional state following the occurrence of AF. This further indicates that personality has a vital impact on the prognosis of AF patients. Possible mechanisms underlying this relationship may involve the activation of the sympathetic nervous system [32], inflammatory pathways [33, 34], and the hypothalamic-pituitary-adrenal axis, as well as the renin-angiotensin-aldosterone system induced by negative emotions [35]. The exact mechanisms, however, require further exploration.

Additionally, our study found that patients with a sanguine personality had the highest scores in both the overall mean AFEQT and AFEQT sub-scale, which are considered effective evaluations for AF treatment [36]. A 2012 Korean study demonstrated that type D personality was an independent predictor of impaired health-related QoL in AF patients [17]. Similar conclusions were drawn by Nina’s team in subsequent research [16]. It is well-known that type D personality is associated with negative emotions such as depressed mood, anger, anxiety, and hostile feelings [37]. However, in our study, AF patients with a sanguine personality, characterized by a sunny and extroverted personality, exhibited the best health-related quality of life. Subjects with a sanguine personality felt less sensitive to symptom severity and demonstrated better endurance in daily activities. Additionally, they reported greater satisfaction with treatment and higher adherence. Conversely, AF patients with negative and introverted emotions (such as phlegmatic and melancholic personalities) were associated with impaired AFEQT. Similarly, previous studies have reported independent impacts of personality types assessed by the EPQ on the prognosis of various diseases. For example, a study conducted by Long Gong suggested that patients with a sanguine personality undergoing total knee arthroplasty had the best postoperative clinical outcomes compared to patients with other personality types. In contrast, choleric patients, who tend to be impulsive, changeable, and touchy, had unexpectedly low satisfaction rates [38]. The present evidence also indicates that patients with a sanguine personality exhibit the most favorable clinical outcomes among the four personality types, and personality types serve as independent risk factors affecting AFEQT in AF patients.

We also observed significant differences in the degree of anxiety and depression among individuals with different personality types. An early study from 1979 reported a link between scores on subdimensions of the EPQ and anxiety disorders [39]. Furthermore, other evidence has demonstrated a positive correlation between anxiety sensitivity and characteristics of several personality disorders, including borderline, histrionic, avoidant, dependent, and passive-aggressive personality disorders [40]. Hagop’s study also reported that patients with a sanguine personality experienced only mild symptoms of depression [41]. Our results align with the conclusions of existing studies, which indicate that anxiety and depression lead to more severe symptoms and poorer AFEQT in AF patients [42]. Moreover, anxiety and depression, as the most common negative emotions, are linked to both impaired QoL [13, 14] and an increased risk of AF [43, 44]. Therefore, psychological counseling is suggested as a necessary intervention for some AF patients with specific personality types.

In this study, we also observed that patients with persistent AF were more prone to depression. This finding is consistent with Alexander’s research, which demonstrated a significant correlation between persistent AF and depression [45]. It can be speculated that persistent AF patients may experience increased concern and worry due to difficulties in restoring normal sinus rhythm.

The limitations of this study are as follows: Firstly, the cross-sectional design of this study limits the ability to establish a definitive causal relationship. And it would be more enlightening if a prospective study was conducted to draw further conclusions about the correlation between personality and AF occurrence. Secondly, sample size of this study is relatively small, and the findings should be further verified in larger multi-center clinical trials. Lastly, the assessment of personality using the EPQ was solely based on self-report measures and may not fully capture the personality types of AF patients. To address this, multiple scales related to personality or QoL should be incorporated.

In conclusion, the results suggest that a sanguine personality is independently linked to better PRO and emotional state in AF patients. These findings highlight the significance of incorporating personality as an integral component of AF patient evaluation and management within clinical practice.

## Data Availability

The dataset analyzed during the current study is not publicly available due to a lack of consent from study participants to do so, but it is available from the corresponding author on reasonable request for researchers who meet the criteria for access to confidential data.

## Abbreviations

PROs: Patient-reported outcomes
AF: Atrial Fibrillation
EPQ: Eysenck Personality Questionnaire
AFEQT: Atrial Fibrillation Effect on Quality of Life
QoL: Quality of Life
GAD-7: General Anxiety Scale
PHQ-9: 9-item Patient Health Questionnaire
CHD: Coronary heart disease
BMI: Body mass index
AADs: Antiarrhythmic drugs
